# Lower Methane Emissions from Yak Compared with Cattle in Rusitec Fermenters

**DOI:** 10.1371/journal.pone.0170044

**Published:** 2017-01-11

**Authors:** Jiandui Mi, Jianwei Zhou, Xiaodan Huang, Ruijun Long

**Affiliations:** 1 School of Life Sciences, State Key Laboratory of Grassland Agro-Ecosystem, International Centre for Tibetan Ecosystem Management, Lanzhou University, Lanzhou, Gansu, China; 2 School of Public Health, Lanzhou University, Lanzhou, Gansu, China; Wageningen University, NETHERLANDS

## Abstract

Globally methane (CH_4_) emissions from ruminant livestock account for 29% of total CH_4_ emissions. Inherited variation about CH_4_ emissions of different animal species might provide new opportunity for manipulating CH_4_ production. Six rumen-simulating fermenters (Rusitec) were set up for this study lasting for 16 d. The diet consisted of forage to concentrate ratio of 50:50 with barley straw as the forage. Treated vessels were supplied with rumen fluid from yak or cattle (3 vessels per animal species). Microbial growth was measured using ^15^N as a marker. The microbial community structure from liquid- and solid-fraction of each vessel was determined based on the 16S rRNA genes targeting both bacteria and archaea with MiSeq platform. CH_4_ yield was lower when the inoculum used from yak than that from cattle (0.26 and 0.33 mmol CH_4_/g dry matter intake, respectively). Lower H_2_ production was observed in Rusitec fermenters with rumen fluid from yak compare with that from cattle (0.28 and 0.86 mmol/d, respectively). The apparent digestibility of neutral detergent fiber, the isovalerate percentage with respect to the total amount of volatile fatty acids, the hydrogen recovery, and the proportion of liquid-associated microbial nitrogen derived from ammonia-nitrogen were higher in Rusitec fermenters incubated with rumen fluid from cattle than that from yak. The relative abundances of methanogens were no difference between two animal species. We hypothesize that more H_2_ production contributes to the higher methane emissions in cattle compare with yak.

## Introduction

Methane (CH_4_) accounts for 11% of total greenhouse gas (GHG) emissions in China. Almost 21% of CH_4_ emissions are due to enteric fermentation in ruminant livestock industry [1, 2]. The Global Warming Potential (GWP) of CH_4_ for a time horizon of 100 years is 28-fold that of CO_2_ [3]. Enteric CH_4_ emissions also represent a 2 to 12% loss of gross energy intake [4]. Many ways to manipulate enteric CH_4_ emissions have been developed, including 4 broad categories: feeds and feeding management, rumen modifiers, genetics and other management strategies [5]. Investigating animals that produce lower CH_4_ might lead to improving livestock systems through modifying rumen fermentation and reducing CH_4_ emissions from other livestock [6–8].

Yak produced 1.7 g of methane /kg W^0.75^ under grazing conditions, which was lower compared with published data about cattle (3.2~4.2 g of methane /kg W^0.75^) [9, 10]. Over 15 million yaks grazed in the Qinghai-Tibetan Plateau account for approximately 90% of the world's total number of yak [11]. Due to the harsh environment in the Qinghai-Tibetan Plateau, which is characterized by hypoxia, strong ultra-violet (UV) radiation, severe cold and deficiencies of forage resources, yak has evolved special abilities on the metabolism of certain nutrients, morphology, and genetics [12–14]. Therefore, yak shows great potential as a “low carbon” animal, which calls for systematic comparative studies of “low-CH_4_ emissions” from yak. However, in a previous study, they conducted an investigation regarding yak without a control group [9]. The level of intake, type and quality of feed, and environmental temperature might contribute to great variation in CH_4_ production [15]. Rumen-simulating fermenters, like the Rusitec fermenters, could be useful tools to evaluate methane emissions from different animals or under different additives treatment because major advantage of this system is the ability to remove fermentation liquid and maintain for relatively long periods of time [16, 17]. The information would be useful using this type of fermenter before conducting expensive and time-consuming *in vivo* study to confirm the difference of methane emissions between yak and cattle. Thus, we performed a comparative study using a Rusitec system to investigate the difference of CH_4_ emissions between yak and cattle under same conditions. The first aim of this study was to confirm if yak is lower CH_4_ producer than cattle under the same conditions. The second aim was to explore the possible link between CH_4_ production and liquid-/solid-associated microbes.

## Materials and Methods

### Animals and treatments

The experiment was conducted from February to April 2014 at the Wushaoling Yak Research Station (37°12.4′N, 102°51.7′E, and altitude 3154m) of Lanzhou University, China. Six castrated male animals (3 cattle; 3 yaks; body weight: 192±12kg) were donor animals of rumen fluid. The yaks (Tianzhu White yak) used in this study were chosen from a local farm at research station. The cattle (Chaidamu Cattle) were bought from Dulan county of Qinghai province, which was grazed all around a year under nature pasture with the *Phragmites communis* as the dominant grass. The use of animals, including their welfare, feeding, and rumen fluid collection, was approved by the Animal Ethics Committee of the Chinese Academy of Lanzhou University (permit number: SCXK Gan 20140215). The sample collection from the animals was handled in accordance with the requirements set forth by the Animal Ethics Procedures and Guidelines of the People's Republic of China. The animals were kept in the metabolism crates in the house with ad libitum access to water and fed 3 kg dry matter/d (approximately 1.5% of body weight), receiving half at 0900 h and the other half at 1800 h during the experiment period. Feed composition was listed in Table 1. Before starting the experiment, animals were familiarized with the environment as well as technical staff feeding to them for 30 days. The average indoor temperature was 6°C, and the relative humidity was 70%. The pens and metabolism crates were cleaned twice a day. The pens were maintained at a good air circulation during experimental study. During experiment period, all animals were kept in a health condition without any medicine treatment. After an adaptation period, the experiment lasted for 20 days, then rumen fluid was taken via the animal’s mouth using a stainless-steel stomach tube attached with a vacuum sampler before morning feeding on the 20^th^ day. After rumen fluid collection, the animals were used for another experiment in our lab. The rumen fluid from three animals with same species was mixed well, maintained in an anaerobic environment using CO_2,_ and used within 1 h for Rusitec study. The mixed rumen fluid from same species was diluted with artificial saliva (550 mL: 450 mL) before dividing 800 mL into each of three fermentation vessels. The pH was controlled between 6.5 and 7.1. Artificial saliva was continuously infused at a rate of 750 mL/d, approximating a dilution rate of 3.9%/h.

**Table 1:** Ingredients and chemical composition of the experimental diet.

Item	Amount
Ingredient, % of DM	
Hulless barley straw	50
Corn	30.5
Wheat red dog	5
Corn starch powder	12.5
Cottonseed oil	0.5
Calcium hydrophosphate	0.5
Commercial premix	0.5
Sodium chloride	0.5
Chemical composition of diet % of DM	
CP	6.45
OM	93.78
Ash	6.22
NDF	67.61
ADF	25.62
metabolizable energy* (MJ/kg DM)	8.35

*Estimated according to the NRC (1985)

DM-dry matter; NDF- neutral detergent fiber; OM-organic matter; ADF-acid detergent fiber.

One trial of 6 Rusitec fermenters was maintained for 16 days. Each fermenter unit had an 800-mL effective volume. The procedure for incubation and the daily operation was consistent with the detailed report by Martínez et al. [18]. All fermenters were filled daily at 0900 h with a 20 g mixture diet of 50: 50 barley straw to concentrate using nylon bags (100μm pore, 5*15 cm) (Table 1). The barley straw was chopped to approximately 3 mm pieces, and the concentrate was ground to pass a 3-mm screen. Before and after feeding, each flask was flushed daily with 1.5 L of gaseous nitrogen to collect the gasses produced from *in vitro* fermentation and to remove the air introduced during feeding, respectively.

The adaptation period of the Rusitec study was from 1 day to 6 day. Digestibility (measured as the substrate disappearance from the bag), pH before feeding, ammonia-N, and VFA were determined on days 7 to 15. The volume of effluent was measured daily, and a 4 mL sample (approximately total 20 mL) was directly frozen (-80°C) for the next analysis of ammonia-N. A 1 mL sample was collected for VFA analysis by diluting 1: 1 in a deproteinizing solution (10% metaphosphoric acid and 0.06% crotonic acid, wt/vol) and stored at -80°C.

Gas was collected from days 7–15 in Guangming gas sampling bags (Zhonghao Guangming Research & Design Institute of Chemical Industry Corporation, Dalian, China), and the volume of fermentation gasses was measured by water displacement. The concentration of CH_4_ was determined using gas chromatography [17]. Hydrogen recovery was calculated according to the equation described by Makkar and Vercoe [17].

On day 11, ^15^NH_4_Cl (99% enriched, Shanghai Engineering Research Centre of Stable Isotope, Shanghai, China) was added into each fermenter at a dose of 2.4 mg of ^15^N to label the NH_3_-N pool instantaneously. It was also necessary to add ^15^NH_4_Cl to the artificial saliva to reach a rate of 4.0 g of ^15^N/kg dietary N.

On days 15 and 16, instead of H_2_SO_4_ solution (20%, vol/vol), which could cause microbe lysis, 5 mL of saturated HgCl_2_ was added to each flask collected liquid effluent. Liquid-associated microbiome pellets (LAM) were obtained from approximately 480 mL effluent by differential centrifugation [19]. The remaining liquid was freeze-dried for analyses of N, non-ammonia nitrogen (NAN), NH_3_-^15^N, ^15^N, and NAN-^15^N enrichment. On days 15 and 16, one part of the nylon bag residues was used to isolate solid-associated microorganisms (SAM); meanwhile, one-fifth of the residue content from each nylon bag was frozen and lyophilized for determination of N, NAN, ^15^N, and NAN-^15^N enrichment. The contents of the nylon bags were treated with a saline solution of 0.1% methylcellulose (0.85% NaCl) at 38°C for 30 min with continuous shaking to elute any attached microorganisms [20]; the residue was re-suspended in a chilled saline solution (same as above) for 24 h after filtering through two layers of nylon cloth (40-μm pore size). The filtrate of the same day was mixed and used to obtain the SAM by centrifugation, as reported by Ranilla and Carro [21]. After treatment, the residue was freeze-dried for the determination of N, NAN, ^15^N, and NAN-^15^N enrichment. On day 16, 300 mg pellets of LAM and SAM were used to extract genome DNA for investigating bacterial and archaea compositions using next generation sequencing of the Miseq Platform. The LAM and SAM pellets were lyophilized and used to analyze the N and ^15^N enrichment. The substrate used in this study was also measured for ^15^N content, the value of which was used for background modifications.

### Analytical procedures

Dry matter, ash, and nitrogen were measured as described by AOAC [22]. Neutral detergent fiber (NDF) was determined according to Van Soest et al. [23] without sodium sulfite and amylase, using VELP SCIENTIFICA FIWE6. The NH_3_-N and VFA concentrations were determined according to Carro and Miller [19]. CH_4_ was measured by GC with FID detector attached by a column of Porapak Q, the condition was same as described by Ding et al. [9]. Preparation of samples for ^15^N analysis was conducted as described by Carro and Miller [19], and ^15^N was analyzed using elementary analyzer-stable isotope ratio mass spectrometers (EA-IRMS, Thermo Delta V advantage and Flash EA 1112 HT).

### DNA extraction and analysis

Genomic DNA was extracted from approximate 300mg (wet weight) LAM and SAM according to the QIAGEN stool kit protocol (51504, Qiagen, Hilden, Germany). DNA was quantified using Nanodrop2000 (Thermo Fisher Scientific Inc., Wilmington, USA) and run on 1.8% agarose gel to confirm DNA integrity. The V4 region of 16S rRNA gene was amplified by PCR using primers 515F-806R as previously described [24, 25]. Reaction conditions consisted of initial denaturation at 95°C for 2 min followed by 25 cycles of denaturation at 95°C for 30 s, annealing at 57°C for 30s, extension at 72°C for 45 s, and a final extension at 72°C for 10 min. Amplicons were quantified using the QubiT PicoGreen dsDNA assay kit (Invitrogen, Grand Island, USA) and mixed at equal masses for a final concentration of approximate 20 ng/μl. The correct size amplicons were recovered from 1.8% agarose gels and purified using QIAquick Gel Extraction Kit (28704, Qiagen, Hilden, Germany) according to the manufacturer’s instructions and quantified using the QubiT PicoGreen dsDNA assay kit (Invitrogen, Grand Island, USA). Purified amplicons were paired-end (2×250 bp) sequenced on an Illumina Miseq platform at Majorbio company (Shanghai, China).

### Sequence analysis

Sequences were processed using the open-source QIIME package, version 1.8 [26]. Data including uncorrectable barcodes, ambiguous bases, and low-quality reads were removed. Operational taxonomic units (OTUs) were picked at 97% identity using Usearch V7.1 [27]. The taxonomic assignment of each OTU was performed against the Greengenes reference taxonomy (Greengenes 13.8). Chao1 and Shannon were used to estimate the community richness and diversity, respectively. Principal coordinate analysis (PCoA) was conducted on the UnWeighted Unifrac distance metric. PICRUSt was used to predict the molecular function of the samples based on 16S rRNA data [28]. The sequence data deposited in EMBL-EBI European Nucleotide Archive (ENA, https://www.ebi.ac.uk/ena/submit/sra) under accession number PRJEB11872.

### Calculations and statistical analysis

The calculations of ammonia-N (mg/d), NAN (mg/d), microbial N flow (mg/d), and proportion of microbial N derived from ammonia-N (%) were performed according to Carro and Miller, [19]. The raw data were collected in Excel 2010. The significance under different fractions was analyzed using SPSS 17.0 with One-Way ANOVA model. The significance value was selected at a 0.05 level. The Tukey-Kramer test was used to evaluate differences between different mean times.

## Results and Discussion

Average values of effluent volume, pH, and substrate digestibility were summarized in Table 2. Because our main aim was to investigate the difference between yak and cattle species, a similar pH under two treatments would maintain a stable environment (Table 2). No differences in effluent volume, apparent disappearance of dry matter (DM), organic matter (OM), and neutral detergent fiber (NDF) were found between the two species (*P*>0.05).

**Table 2:** Effect of animal species on the level of the pH and amount of apparent disappearance under same low nitrogen diet in Rusitec fermenters.

Item	Cattle	Yak	SEM	*P*-value
Effluent, L/d	0.775	0.781	00024	0.776
pH before feeding	6.84	6.82	0.02	0.383
Apparent ruminal digestibility, %				
DM	42.2	40.9	1.10	0.245
NDF	68.9	69.6	1.95	0.69
OM	41.9	40.4	1.09	0.192

DM-dry matter; NDF- neutral detergent fiber; OM-organic matter.

To our knowledge, this is the first study in which yak and cattle species were compared using Rusitec fermenters to investigate differences in methane production. The total amounts of gas and methane were higher in fermenters used rumen fluid from cattle, showing 27% and 32% increases in production, respectively (Table 3). However, the results were still lower than those of other studies in Rusitec fermenters [18, 29]. Under the same diet conditions, the donor animal was the main reason for the differences in gas and methane production [30].

**Table 3:** CH_4_ production and volatile fatty acids (VFA) in Rusitec fermenters (means for the whole experimental period).

Item	Cattle	Yak	SEM	*P*-value
Total gas production, mmol/d	50.6	39.7	3.990	0.010
CH_4_, mmol/d	6.2	4.7	0.602	0.023
CH_4_ per g DM, mmol/g DM intake	0.33	0.26	0.026	0.032
CH_4_ per g OM intake, mmol/g OM intake	0.35	0.27	0.026	0.034
CH_4_ per g DM, mmol/g digestible DM	1.11	0.84	0.109	0.019
CH_4_ per g DM, mmol/g digestible OM	0.85	0.68	0.082	0.049
H_2_, mmol/d	0.86	0.28	0.099	0.017
Total VFA production, mmol/d	38.1	46.6	5.420	0.127
Molar proportion (mol/100 mol)				
Acetate	50.1	49.4	0.864	0.424
Propionate	32.3	31.8	0.994	0.650
Butyrate	12.1	13.7	0.468	0.002
Isobutyrate	0.253	0.258	0.091	0.956
Valerate	3.81	3.76	1.012	0.965
Isovalerate	1.46	1.06	0.158	0.019
Acetate: Propionate	1.55	1.57	0.062	0.681
H recovery, %	85.56	73.91	3.703	0.004

DM-dry matter; OM-organic matter.

Total volatile fatty acids (VFA) and VFA profiles (acetate, propionate, isobutyrate, valerate, and acetate to propionate ratio) showed no observable differences between the two species (*P*>0.05, Table 3). Butyrate and isovalerate were affected by the two species. Butyrate was higher with yak species than cattle species. In contrast, isovalerate was lower in yak species (*P*<0.05, Table 3). Previously, the propionate, butyrate, and acetate to propionate ratios were affected by the donor animals of cow and sheep [30]. The different results could be due to several reasons, including the microbial composition of the rumen ecosystem [31]. Hydrogen recovery was higher in cattle species than yak species, meaning that more hydrogen was used by microbes in cattle species (*P*<0.05, Table 3).

Microbial growth is one of the key measurements in an in *vitro* system because of the important roles played by microbes in host health and energy supply [32]. A suitable marker is very important for differentiating microbial nitrogen from different parts of feed degradation. In the present study, ^15^N was selected as marker due to its accuracy [33]. As shown in Table 4, no differences were observed in ammonia production, daily production of non-NH_3_ N, flows of microbial nitrogen, and SAM nitrogen derived from ammonia-N (*P*>0.05). Although ^15^N enrichment of NH_3_-N and LAM were lower in cattle species than in yak species, the proportion of LAM nitrogen derived from ammonia-N was greater for cattle species (*P*<0.05). However, a lower enrichment of the SAM was found in both cattle and yak species compared with the LAM, which was in agreement with the previous comparative report regarding Merino sheep and Rusitec fermenters [32]. The ^15^N enrichment was lower in the SAM than in the LAM (the mean values were 0.5210 and 1.5298 atom% in excess in cattle species, respectively, and 0.5842 and 1.7620 atom% in excess in yak species, respectively). The LAM was located in free rumen fluid and SAM was loosely/tightly attached to feed particles and associated with feed surfaces, where the ammonia concentration may be lower on the surfaces of feed particles than in the rumen fluid. As a consequence of the differences in ^15^N enrichment between the LAM and the SAM, the percent of microbial nitrogen derived from NH_3_-N was lower in the SAM than in the LAM in both cattle and yak species (Table 4). The results of the nitrogen differences in the SAM and LAM suggested that these two different parts should be taken into account to obtain a more reliable result in determining microbial protein production in further studies.

**Table 4:** Nitrogen content and daily production of ammonia-N, NAN, and microorganisms, the proportion of microbial N derived from ammonia in Rusitec fermenters.

Item	cattle	yak	SEM	*P*-value
Ammonia-N, mg/d	59.4	48.8	7.119	0.178
NAN, mg/d	96.7	102.2	3.984	0.204
Microbial N flow,				
Total microorganisms, mg/d	72.1	76.4	4.044	0.338
LAM, mg/d	35.6	34.4	3.422	0.737
SAM, mg/d	36.5	42.0	5.021	0.318
SAM % of total	50.6	54.5	4.750	0.444
N content of LAM, mg/g DM	59.8	62.7	2.786	0.394
N content of SAM, mg/g DM	62.4	64.8	0.973	0.114
^15^N enrichment, atoms % excess				
LAM	1.5298^a^	1.7620^a^	0.053	0.015
SAM	0.5210^b^	0.5842^b^	0.043	0.276
Ammonia-N	3.2416	4.0361	0.067	0.001
Proportion of microbial N derived from ammonia-N, %				
LAM	47.2^a^	43.7^a^	1.25	0.049
SAM	16.1^b^	14.5^b^	1.41	0.367

DM-dry matter; LAM-liquid-associated microorganisms; SAM- solid-associated microorganisms; NAN- non-ammonia nitrogen.

^a-b^Means within a column without common superscript letters differ between LAM and SAM (*P* < 0.05).

**Fig 1:**
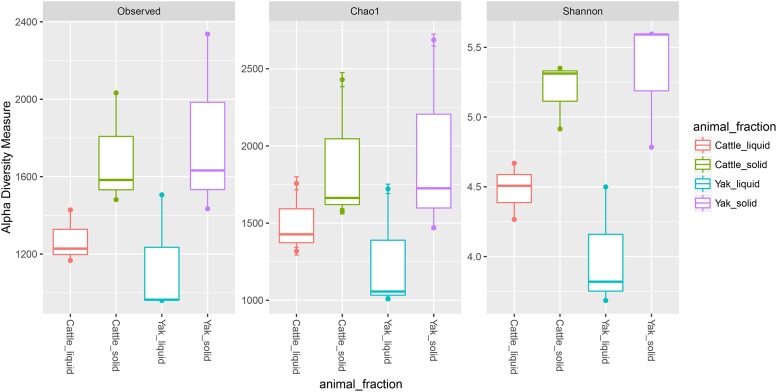
Changes in alpha diversity values among different groups.

**Fig 2:**
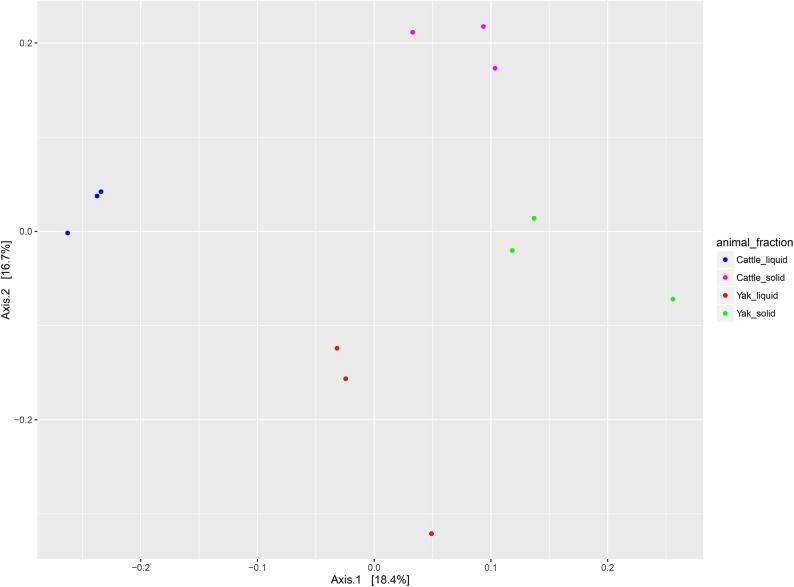
Principal coordinate analysis (PCoA) of the microbiota community based on UnWeighted Unifrac distance.

**Fig 3:**
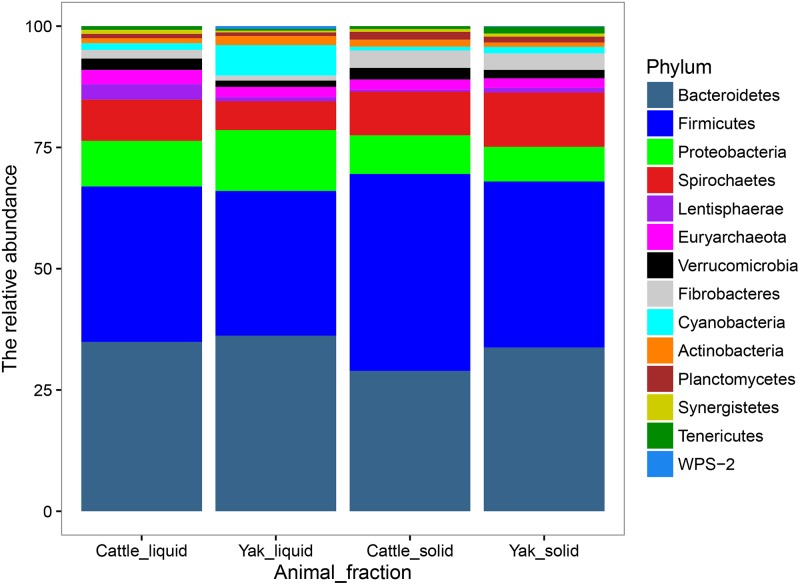
The impact of different fractions in Rusitec fermenters on the microbiota composition.

Considering the observed differences in gas production, digestibility, and microbial growth, the microbial composition may be the main reason for these results. Thus, we isolated genomic DNA from the LAM and SAM. A culture-independent method, next generation sequencing technology using Miseq-250 platform, was used to assess the microbial community structures. After quality control, a total of 962,074 reads were obtained for the V4 16S rRNA sequences, with an average of 80, 172 ± 6392 (SD) per sample. The average length of the sequence reads was 273 bp. The number of OTUs observed in this study reached 9, 794 based on a similarity threshold of 97% at species level. No differences were found for all alpha-diversity index between yak and cattle species from same fraction in Rusitec fermenters (*P*>0.05, Fig 1), which was consistent with the previous comparison study of low and high methane production cattle [34]. However, alpha diversity index of the solid-associated microbes from the same animal group was higher than that of liquid-associated microbes. The lowest values of alpha diversity were found in the rumen liquid-associated microbes of yak species. The PCoA analysis using the UnWeighted Unifrac metric indicated that the samples clustered according to the different parts in Rusitec fermenters with different animals (Fig 2). In total, 14 phyla were identified as being distributed across all the samples in Rusitec fermenters (Fig 3). *Bacteroidetes*, *Firmicutes*, *Spirochaetes*, and *Proteobacteria* were dominant phyla, regardless of the group (Fig 3), but their proportions varied among the groups, as has been found by many others regarding rumen microbe studies [34]. Six phyla were affected by different animal fractions (*Proteobacteria*, *Spirochaetes*, *Lentisphaerae*, *Verrucomicrobia*, *Fibrobacteres* and *Cyanobacteria*) (*P*<0.05, S1 Table). The phylum *Proteobacteria* was the highest in the LAM and lowest in the SAM of yak species. *Cyanobacteria*, *Spirochaetes*, *and Lentisphaerae* were the highest in LAM of yak species, SAM of yak species, and LAM of cattle species, separately. The *Fibrobacteres* was higher in SAM parts than in LAM regardless of different animals. Because *Fibrobacteres* was most important fiber degradation microbiomes [35]. *Verrucomicrobia* was higher in cattle than yak species regardless of the fractions.

**Fig 4:**
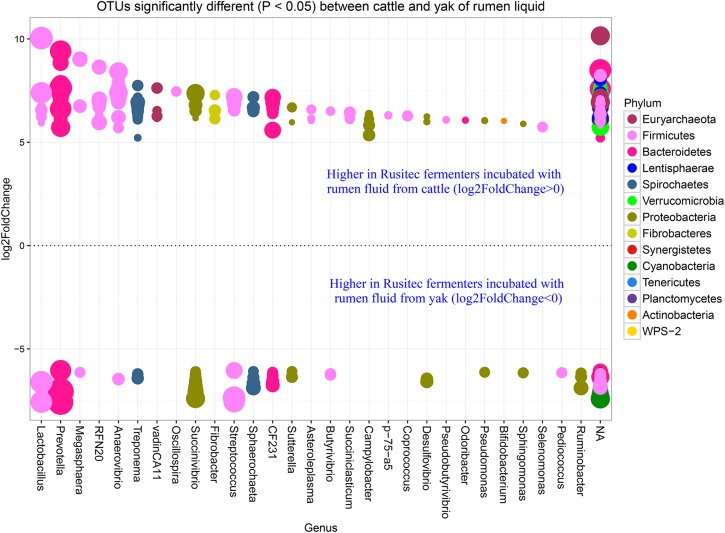
OTUs counts significantly different (*P* < 0.05) between cattle and yak of rumen liquid. The size of the dot represents the value of the OTU, the higher value of the OTU number, the bigger size of the dot. The P value was modified by p.adjust value.

**Fig 5:**
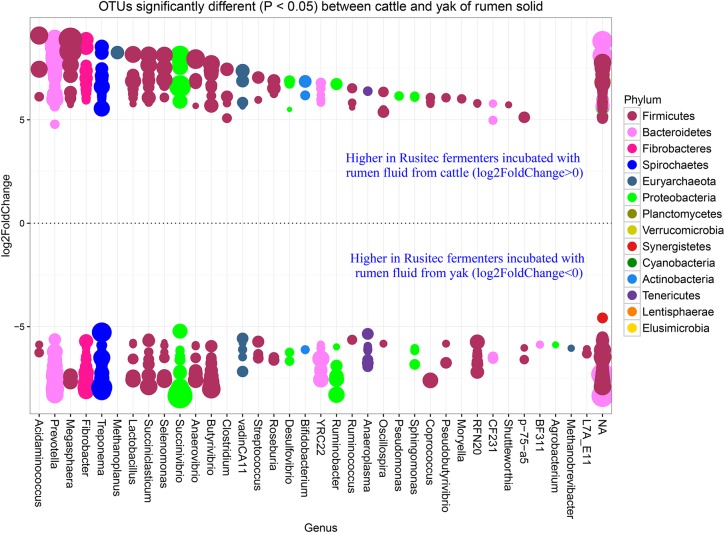
OTUs counts significantly different (*P* < 0.05) between cattle and yak of rumen solid. The size of the dot represents the value of the OTU, the higher value of the OTU number, the bigger size of the dot. The P value was modified by p.adjust value.

**Fig 6:**
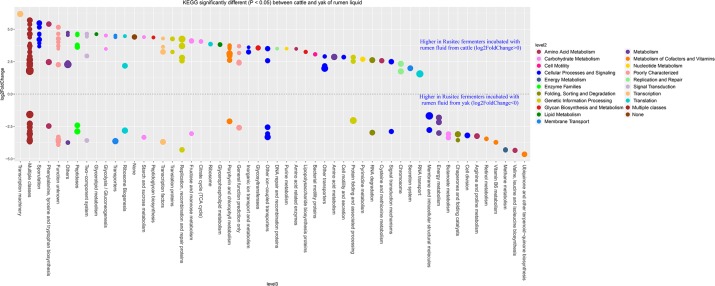
Predicted KEGG functions count significantly different (*P* < 0.05) between cattle and yak of rumen liquid. The size of the dot represents the value of the function genes, the higher value of the function genes number, the bigger size of the dot. The P value was modified by p.adjust value. The predicted data was generated by PICRUSt, the analysis and figure were conducted by R (3. 2. 3).

**Fig 7:**
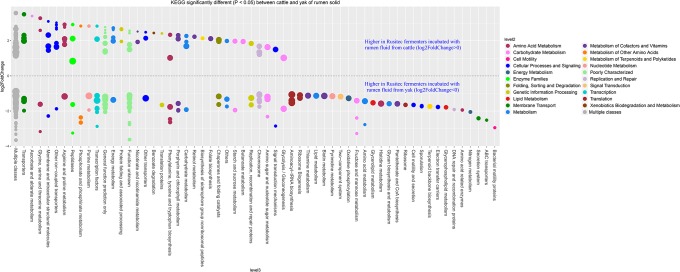
Predicted KEGG functions count significantly different (*P* < 0.05) between cattle and yak of rumen solid. The size of the dot represents the value of the function genes, the higher value of the function genes number, the bigger size of the dot. The P value was modified by p.adjust value. The predicted data was generated by PICRUSt, the analysis and figure were conducted by R (3. 2. 3).

In our study, yak produced less methane than cattle with the same diet in *vitro* semi-continuous culture system (Rusitec). Thus, we want to know the difference at genus level between yak and cattle with the same fractions. The fold2changes were showed in Figs 4 and 5. *RFN20*, *vadinCA11*, *Fibrobacter*, *Asteroleplasma*, *Succiniclasticum*, *Campylobacter*, *p−75−a5*, *Coprococcus*, *Pseudobutyrivibrio*, *Odoribacter*, *Bifidobacterium*, and *Selenomonas* were higher in LAM of cattle and only *Pediococcus* and *Ruminobacter* were higher in LAM of yak species. *Methanoplanus*, *Clostridium*, *Pseudomonas*, *Moryella*, and *Shuttleworthia* were higher in SAM of cattle species and *BF311*, *Agrobacterium*, *Methanobrevibacter*, and *L7A_E11* were higher in SAM of yak species. Higher abundances of bacteria as H_2_ producers existed in cattle species, such as *Coprococcus*, *Succiniclasticum*, and *Clostridium* [36]. However, the dominant methanogen genus *Methanobrevibacter* was higher in the SAM of yak than cattle species [37]. While the new order of methanogen *Methanomassiliicoccales* (Genus *vadinCA11*) was higher in LAM of cattle species. The difference between cattle and yak species was not linked regularly with the methane production. That might be the reason of more diversity of bacteria existed in the cattle fermenters, which contributed to the substrate needed by methanogen *Methanomassiliicoccales*. There were a few higher abundance bacteria in the yak species, which would produce less H_2_, in agreement with the less H_2_ production of yak compare with cattle (Table 3). In order to confirm the function difference between yak and cattle species, we used PICRUSt to predict the function of the microbiomes based on 16S RNA sequenced data (Figs 6 and 7). The result showed that many functions were different between two species (Figs 6 and 7), however, we just focused on the ones about nutrition and energy metabolism to discuss. Energy metabolism, vitamin B6 metabolism, and methane metabolism were higher in LAM of yak species. The higher methane metabolism function in LAM of yak might be the consequence of less diversity of microbiome existed (Fig 1). That difference might be the consequence of the *in vitro* study limits. In future, *in vivo* study should be conducted to compare the methane difference between yak and cattle. However, the predicted functions involved in sugar, fat, protein and amino acid were higher in LAM of cattle species. Vitamin B6 metabolism was higher in the yak species, which might be the consequence of the higher relative abundance of phylum *Cyanobacteria* [38]. Twenty-six predicted functions were higher in SAM of yak than cattle species, indicating more microbiome pathways existed to help yak to adapt the harsh environment. The further study should be conducted to confirm this hypothesis.

In conclusion, the results showed that yak was potential “low carbon” ruminant. The different microbe compositions correlated with methane emissions. The data might be used to manipulate or provide useful information to reduce the environmental effects of other ruminants.

## Supporting Information

S1 Table The microbial compostions at phylum level. a-c Means within a row without common superscript letters differ under different treatmens (*P* < 0.05).
(XLSX)
